# Quality of IVM ovarian tissue oocytes: impact of clinical, demographic, and laboratory factors

**DOI:** 10.1007/s10815-024-03234-2

**Published:** 2024-09-30

**Authors:** Maria Kashutina, Lilia Obosyan, Ekaterina Bunyaeva, Yury Zhernov, Anastasia Kirillova

**Affiliations:** 1 Russian University of Medicine, Moscow, Russia; 2 Loginov Moscow Clinical Scientific and Practical Center, Moscow, Russia; 3 National Research Centre for Therapy and Preventive Medicine, Moscow, Russia; 4 I.M. Sechenov First Moscow State Medical University, Moscow, Russia; 5 National Medical Research Center for Obstetrics, Gynecology and Perinatology Named After V.I.Kulakov, Moscow, Russia; 6 A.N. Sysin Research Institute of Human Ecology and Environmental Hygiene, Moscow, Russia; 7 Fomin Clinic, Moscow, Russia; 8 Royal Women’s Hospital, Melbourne, Australia; 9 University of Melbourne, Melbourne, Australia

**Keywords:** Ovarian tissue oocytes, IVM, Fertility preservation, Oocyte dysmorphism, Oocyte competence, Oocyte quality

## Abstract

**Purpose:**

To determine how clinical, demographic, and laboratory characteristics influence ovarian tissue oocyte quality.

**Methods:**

Immature cumulus-oocyte complexes were isolated from removed ovaries and cultured for 48–52 h in either monophasic standard or biphasic CAPA media for fertility preservation. A total of 355 MII oocytes from 53 patients were described for intracytoplasmic and extracytoplasmic anomalies. Multiple clinical, laboratory, and demographic characteristics were analyzed. Statistically significant differences between independent groups in qualitative variables were identified using Pearson’s χ^2^ and Fisher’s exact tests. The diagnostic value of quantitative variables was assessed using the ROC curve analysis. Factors associated with the development of dysmorphism, taking patient age into account, were identified using the binary logistic regression analysis.

**Results:**

Dysmorphisms were observed in 245 oocytes (69.0%), with a median number of dysmorphisms of 2. Oocyte dysmorphisms were found to be 2.211 times more likely to be detected in patients with ovarian cancer, while the presence of dark-colored cytoplasm was associated with gynecologic surgery in the anamnesis (*p* = 0.002; OR 16.652; 95% CI, 1.977–140.237; Cramer’s *V* 0.187). Small polar bodies developed 2.717 times more often (95% CI, 1.195–6.18) in patients older than 35. In the case of ovarian transportation on ice at 4 ℃, the chances of development of cytoplasmic granularity increased 2.569 times (95% CI, 1.301–5.179). The use of biphasic CAPA IVM media contributed to a decrease in the probability of large polar body formation (*p* = 0.034) compared to the standard monophasic IVM media.

**Conclusions:**

Both patients’ characteristics and laboratory parameters have an impact on the quality of IVM ovarian tissue oocytes.

**Supplementary Information:**

The online version contains supplementary material available at 10.1007/s10815-024-03234-2.

## Background

Ovarian tissue oocytes in vitro maturation (OTO IVM) is a method of oncofertility based on the in vitro culture of immature cumulus-oocyte complexes isolated from the ovarian medulla. This method gains hope for women with radical ovariectomy to have their biological children, and several live births after OTO IVM programs have proven the validity of this method [1–3]. However, this data is limited, and our knowledge of the competence of OTO to fertilization, support embryonic development, and successful pregnancy progression is still not complete, which makes it difficult to consult patients on their chances of live birth after OTO IVM.

While we can make some estimates of the blastocysts competency obtained after controlled ovarian stimulation [4–6], especially after genetic testing [7–11], it is more difficult to predict the competence of oocytes in general. The quality of oocytes is primarily assessed by their morphological characteristics before and after fertilization. Usually, oocyte morphological abnormalities also known as dysmorphisms are classified as intracytoplasmic and extracytoplasmic anomalies. Extracytoplasmic abnormalities include shape abnormalities (irregular shape or ovoid shape), zona pellucida (ZP) abnormalities (dark or thick ZP), and perivitelline space (PVS) abnormalities (large PVS and PVS granularity). Intracytoplasmic abnormalities include different types and degrees of cytoplasmic granulations; color variations (dark-colored cytoplasm, slightly, or excessive whole/centrally located granulation); and the presence of refractile bodies, smooth endoplasmic reticulum (SER), or vacuolization in the ooplasm [12].

It is well known that the competency of an embryo for implantation and pregnancy progression is correlated with the morphological and structural maturity of oocytes [13–17]. For instance, oocyte fertilization is negatively impacted by certain oocyte dysmorphisms including a large first polar body, the perivitelline space (PVS) size, and the presence of vacuoles or refractile bodies [14, 16, 18–21].

A significant reduction in the inner cell mass (ICM) quality by 15% and a 50% decline in trophectoderm (TE) quality was associated with large PVS in oocytes. SER also affects embryo quality and development [22–26] as well as pregnancy rates [25, 27, 28]. SER contributed to a 35% reduction in inner cell mass (ICM) quality, a 67% decline in the probability of pregnancy, and a 20% increased risk of miscarriage [22]. This may be associated with the fact that the presence of SER may negatively affect the oscillation of calcium concentration in the cytoplasm, which is essential for successful fertilization [23, 28, 29]. SER is considered one of the worst predictors of oocyte quality, and The Istanbul Consensus on embryo assessment even recommended refraining from utilizing SER oocytes in assisted reproductive technology (ART) treatments [30].

Visualization and granulation of the cytoplasm are other markers of poor oocyte quality. It has been demonstrated that the presence of these dysmorphisms affects the fertilization rate, associated with a lower potential for good quality blastocysts and pregnancy outcome, while the cryo survival of embryos derived from such oocytes was almost halved [14, 15, 19, 31–39].

Perivitelline space abnormalities (large PVS and the presence of debris in PVS) can be detrimental to embryo development, implantation, and pregnancy rates [40, 41]. According to the Setti meta-analysis [21], the probability of oocyte fertilization is significantly reduced in the presence of large PVS, although the impact on implantation and pregnancy rates remains unclear. Despite the small number of cycles examined, coarse granules in PVS were inversely correlated with the rate of implantation and pregnancy [42]. Fertilization, implantation, and pregnancy rates were negatively affected by ovoid oocytes and oocytes with zona pellucida abnormalities. Oocytes with dark ZP might have some impact on the outcome of ART treatment, including reduced fertilization rates, implantation, clinical pregnancy, and live birth rates [43, 44]. Ovoid oocytes exhibit delays in the early stages of development before implantation, potentially attributed to a decreased occurrence of cell-to-cell contacts [22, 45].

Nonetheless, certain studies have indicated that there are no significant differences in fertilization rates and embryo quality between normal and oval-shaped oocytes [46] and that ovoid-shaped oocytes can result in successful pregnancies [47]. In contrast, several studies have shown that oocyte shape anomalies, i.e., non-spherical shapes, do not appear to affect the fertilization rate, embryo quality, and implantation or pregnancy rates except for the ovoid oocytes [16, 48–50].

According to the literature, about 60–80% of human oocytes have one or more abnormal morphological characteristics [12, 14, 33, 34, 51]. However, these studies were carried out on oocytes retrieved after controlled ovarian stimulation. Little data is available on the quality of in vitro matured oocytes and the factors that might influence them. In this study, we explored the influence of both patient and laboratory characteristics on oocyte dysmorphism formation.

## Methods

### Study population

An observational retrospective single-center uncontrolled study was conducted from October 2018 to February 2021 and included data on all patients who sought OTO IVM fertility preservation programs during this period and got at least 1 mature oocyte after OTO IVM (*n* = 53 women; mean age 31 years; age range 14–43 years). Indications for OTO IVM were endometrial cancer (EC) (*n* = 15), ovarian cancer (OC) (*n* = 13), cervical cancer (CC) (*n* = 10), borderline ovarian tumors (BOTs) (*n* = 6), breast cancer (BC) (*n* = 5), Hodgkin lymphoma (HL) (*n* = 3), and Ewing sarcoma (ES) (*n* = 1). The patients did not receive ovarian stimulation before the surgery. This study was approved by the Ethics Committee (protocol no. 11 from 13.12.2018). A written informed consent was obtained from all patients.

### Selection of factors

The database was formed based on data from the patient’s medical records. Among the clinical and anamnestic data, such indicators as age (the patients were divided into three groups—less than 18 years old, 18–35 years old, and 35 years old and older), body mass index (BMI) (according to BMI level, the patients were divided into 4 groups—underweight, BMI < 18.5 kg/m^2^; normal, BMI = 18.5–24.9 kg/m^2^; overweight, BMI = 25.0–29.9 kg/m^2^; obesity, BMI ≥ 30.0 kg/m^2^), oral contraceptive use, and smoking habit were taken into account. Among fertility factors, the level of the antimullerian hormone (AMH) as a marker of ovarian reserve, the type of infertility (patients were divided by this indicator into three groups—pregnancy was not planned by a patient, primary infertility, or secondary infertility), menstrual cycle (MS) length in days, oral contraceptive use, number of antral follicles, and number of pregnancies and live births were recorded. Indicators related to cancer were considered, such as the type and stage of cancer, the presence of malignant cells in ovaries, the inheritance of oncological disease, and the fact of undergoing and the number of chemotherapy courses, as well as the number of laparoscopic surgical interventions on the pelvic organs in the anamnesis. The IVM media (standard monophasic or biphasic CAPA), temperature (37 °C or 4 °C), and duration of ovary transportation in minutes were studied as laboratory indicators.

### Ovarian tissue transportation and OTO IVM

Full ovaries were transported on ice at 4 ℃ or 37 ℃ in a transport container**.** Immature cumulus-oocyte complexes were isolated from a medulla tissue using the scalpels and 21-gauge needles connected to a syringe. COCs were cultured in groups in monophasic standard IVM or biphasic CAPA IVM medium as previously described [52]. All oocytes were denuded by SynVitro Hyadase (Origio, CooperSurgical) and then immediately vitrified or fertilized via ICSI.

### Dysmorphisms evaluations

**Fig. 1:**
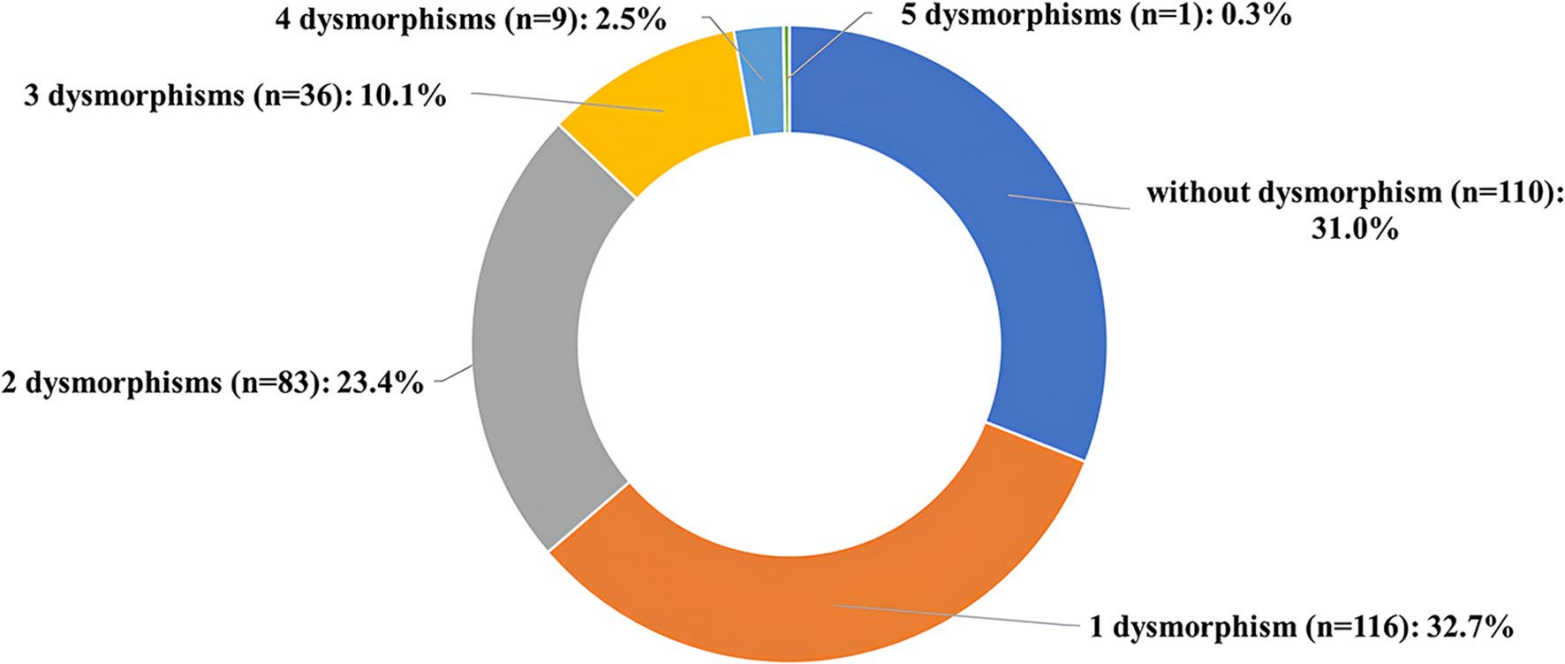
Distribution of oocytes according to the number of detected dysmorphisms

Images of each oocyte were taken with the Olympus IX73 microscope with OCTAX EyeWare 2.2.2.318 software (Vitrolife, Gothenburg, Sweden) right after oocyte denudation. The image of each mature oocyte was thoroughly analyzed by one scientist on the presence of any intracytoplasmic (vacuoles, granularity, dark color of cytoplasm, smooth endoplasmic reticulum clusters, uneven oolemma, refractile body) or extracytoplasmic (oval-shape ZP, debris in the perivitelline space, large perivitelline space, thick zona pellucida, large polar body, small polar body, fragmented polar body) abnormalities (Fig. 1).

### Statistical analyses

Statistical analysis was performed using the program BM SPSS 27.0. When working with quantitative variables, we checked for normality of data distribution. The normality of the distribution was assessed by the kurtosis and skewness indices, and by the Lilliefors-corrected Kolmogorov–Smirnov test. The level of *p* > 0.05 was considered critical. If it was reached, the data distribution was considered normal. The analyzed quantitative features had a distribution different from normal; therefore, when describing them, the median (Me) and interquartile range [Q1–Q3] were calculated. Qualitative indicators are presented as absolute values and percentages. The statistical significance of differences between the analyzed percentages in independent groups was assessed using Pearson’s χ2 criterion and Fisher’s exact test. If statistically significant differences were found, the strength of the association between features (size effect) was assessed using Cramer’s *V* value according to the recommendations of Rea and Parker (Table 1).

**Table 1:** Interpretation of Cramer’s *V* values (Cramer’s *V*) according to Rea and Parker recommendations

Cramer’s *V* value	Strength of association
< 0.1	Negligible
0.1– < 0.2	Weak
0.2– < 0.4	Moderate
0.4– < 0.6	Relatively strong
0.6– < 0.8	Strong
0.8–1.0	Very strong

ROC analysis was performed to assess the diagnostic significance of quantitative variables in predicting the development of dysmorphisms. The binary logistic regression method was used to calculate the odds ratio (OR) for quantitative variables that showed statistically significant association with the studied variables, as well as to build two-factor age-adjusted prognostic models (AOR). In two-factor models, the association of factors with outcome was assessed using the enter-forced inclusion method. Factors included in the regression models were analyzed based on the OR and 95% confidence interval (CI) values. Predictors were considered significant at the *p* < 0.05 level.

## Results

**Fig. 2:**
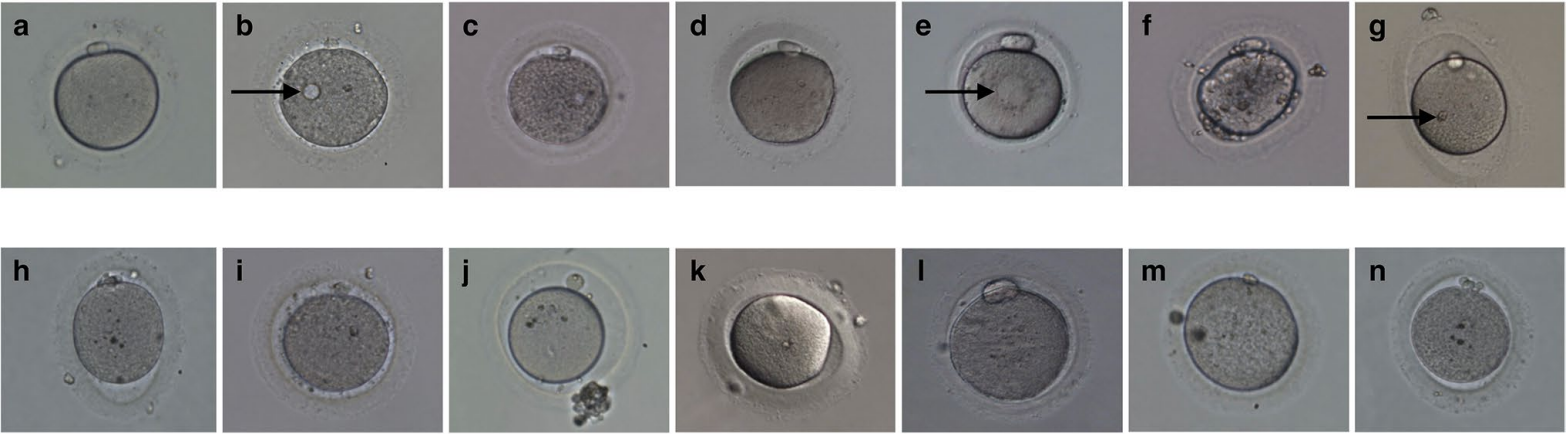
Examples of dysmorphisms present in ovarian tissue oocytes. Each oocyte might have several dysmorphisms. Arrows pointing at the dysmorphism: **a** normal oocyte, **b** oocyte with a vacuole, **c** oocyte with granular cytoplasm, **d** oocyte with dark-colored cytoplasm, **e** oocyte with smooth endoplasmic reticulum cluster, **f** oocyte with uneven oolemma, **d** oocyte with a refractile body, **h** oocyte with ovoid zona pellucida, **i** oocyte with debris in the perivitelline space, **j** oocyte with large perivitelline space, **k** oocyte with thick zona pellucida, **l** oocyte with a large polar body, **m** oocyte with a small polar body, **n** oocyte with a fragmented polar body

We analyzed data on 355 MII oocytes (Me 4.0 [2.0–9.0] oocytes per patient) obtained from 53 patients. Dysmorphisms were observed in 245 oocytes (69.0%), with a median number of dysmorphisms of 2.0 [1.0–2.0] per oocyte (Fig. 2). A total of 129 (52.7%) oocytes showed more than one dysmorphism. The dysmorphisms of mature OTO after IVM were analyzed in detail (Table 2). The most frequently detected dysmorphisms were large perivitelline space, fragmented polar body, and cytoplasmic granularity (44.9%, 30.6%, and 29.4%, respectively).

**Table 2:** Morphological characteristics in vitro matured MII ovarian tissue oocytes

Type of dysmorphisms	Frequency of dysmorphisms (*n* = 245)
Intracytoplasmic	
Vacuoles, *n* (%)	33 (13.5%)
Granularity, *n* (%)	72 (29.4%)
Dark color of cytoplasm, *n* (%)	7 (2.9%)
Smooth endoplasmic reticulum, *n* (%)	8 (3.3%)
Uneven oolemma, *n* (%)	9 (3.7%)
Refractile body, *n* (%)	1 (0.4%)
Extracytoplasmic	
Ovoid ZP, *n* (%)	14 (5.7%)
Debris in the perivitelline space, *n* (%)	52 (21.2%)
Large perivitelline space, *n* (%)	110 (44.9%)
Thick ZP, *n* (%)	7 (2.9%)
Large polar body, *n* (%)	9 (3.7%)
Small polar body, *n* (%)	34 (13.9%)
Fragmented polar body, *n* (%)	75 (30.6%)

*ZP* zona pellucida

One of the aims of this study was to find an association between the presence of certain types of dysmorphisms and demographic, clinical, and laboratory characteristics (Table 3). We found that ovarian cancer as a diagnosis increased the odds of detecting oocyte dysmorphisms by a factor of 2.211 (95% CI, 1.032–4.738; *p* = 0.04). Evaluation of the association of the compared indicators using Cramer’s *V* demonstrated that this association is of weak strength (*V* = 0.112). The age-adjusted model also confirmed the observed patterns (OR = 2.352; 95% CI 1.075–5.149; *p* = 0.032). The other characteristics showed no statistically significant associations (*p* > 0.05).

**Table 3:** Associations of demographic, clinical, and laboratory characteristics with formation of oocyte dysmorphisms

Characteristics			Abs. number of MII oocytes (% from all)	Abs. number of MII oocytes with dysmorphisms (% in a group)	Abs. number of MII oocytes without dysmorphisms (% in a group)	*p*-value	
Age, years (*n* = 347)	18–35		270 (77.8)	183 (67.8)	87 (32.2)	*p* = 0.655	
	< 18		21 (6.1)	16 (76.2)	5 (23.8)		
	≥ 35		56 (16.1)	40 (71.4)	16 (28.6)		
	*p*-value for trend		347 (100.0)	-	-	*p* = 0.688	
BMI (*n* = 329)	18.5–24.9		151 (45.9)	106 (70.2)	45 (29.8)	*p* = 0.973	
	< 18.5		55 (16.7)	37 (67.3)	18 (32.7)		
	25.0–29.9		68 (20.7)	48 (70.6)	20 (29.4)		
	≥ 30.0		55 (16.7)	39 (70.9)	16 (29.1)		
	*p*-value for trend		329 (100.0)	-	-	*p* = 0.98	
Smoking (*n* = 347)	No		337 (97.1)	233 (69.1)	104 (30.9)	*p* = 0.509	
	Yes		10 (2.9)	6 (60.0)	4 (40.0)		
Oral contraceptive use (*n* = 347)	No		273 (78.7)	185 (67.8)	88 (32.2)	*p* = 0.391	
	Yes		74 (21.3)	54 (73.0)	20 (27.0)		
The day of the MC (*n* = 322)	*p*-value for trend		322 (100.0)	-	-	*p* = 0.273	
No. of pregnancies (*n* = 347)	*p*-value for trend		347 (100.0)	-	-	*p* = 0.901	
No. of live birth (*n* = 347)	*p*-value for trend		347 (100.0)	-	-	*p* = 0.823	
AMH level (*n* = 169)	*p*-value for trend		169 (100.0)	-	-	*p* = 0.555	
Fertility status (*n* = 347)	Not infertility		197 (56.8)	135 (68.5)	62 (31.5)	*p* = 0.897	
	Primary infertility		131 (37.8)	90 (68.7)	41 (31.3)		
	Secondary infertility		19 (5.5)	14 (73.7)	5 (26.3)		
IVM medium (*n* = 347)	Standard		234 (67.4)	160 (68.4)	74 (31.6)	*p* = 0.772	
	CAPA		113 (32.6)	79 (69.9)	34 (30.1)		
Main diagnosis (*n* = 347)	BOT	No	324 (93.4)	226 (69.8)	98 (30.2)	*p* = 0.243	
		Yes	23 (6.6)	13 (56.5)	10 (43.5)		
	CC	No	290 (83.6)	204 (70.3)	86 (29.7)	*p* = 0.183	
		Yes	57 (16.4)	35 (61.4)	22 (38.6)		
	EC	No	194 (55.9)	131 (67.5)	63 (32.5)	*p* = 0.541	
		Yes	153 (44.1)	108 (70.6)	45 (29.4)		
	BC	No	316 (91.1)	220 (69.6)	96 (30.4)	*p* = 0.416	
		Yes	31 (8.9)	19 (61.3)	12 (38.7)		
	OC	No	298 (85.9)	199 (66.8)	99 (33.2)	*p* = 0.04	
		Yes	49 (14.1)	40 (81.6)	9 (18.4)		
	HL	No	320 (92.2)	221 (69.1)	99 (30.9)	*p* = 0.83	
		Yes	27 (7.8)	18 (66.7)	9 (33.3)		
	ES	No	340 (98.0)	233 (68.5)	107 (31.5)	*p* = 0.442	
		Yes	7 (2.0)	6 (85.7)	1 (14.3)		
Stage of Ovarian Cancer (*n* = 49)	IA		5 (10.2)	5 (100.0)	0 (0.0)	*p* = 0.261	
	IB		9 (18.4)	5 (55.6)	4 (44.4)		
	IC		11 (22.4)	10 (90.9)	1 (9.1)		
	IIIA		2 (4.1)	2 (100.0)	0 (0.0)		
	IIIC		22 (44.9)	18 (81.8)	4 (18.2)		
Presence of malignant cells in ovaries (*n* = 347)	No		275 (79.3)	186 (67.6)	89 (32.4)	*p* = 0.33	
	Yes		72 (20.7)	53 (73.6)	19 (26.4)		
Inheritance of oncological disease (*n* = 347)	No		270 (77.8)	181 (67.0)	89 (33.0)	*p* = 0.166	
	Yes		77 (22.2)	58 (75.3)	19 (24.7)		
PCTs (*n* = 347)	No		299 (86.2)	206 (68.9)	93 (31.1)	*p* = 0.984	
	Yes		48 (13.8)	33 (68.8)	15 (31.3)		
	*p*-value for trend		347 (100.0)	-	-	*p* = 0.994	
No. of AF (*n* = 260)	*p*-value for trend		260 (100.0)	-	-	*p* = 0.268	
Gynecological surgeries in anamnesis (343)	No		248 (72.3)	167 (67.3)	81 (32.7)	*p* = 0.255	
	Yes		95 (27.7)	70 (73.7)	25 (26.3)		
	*p*-value for trend		343 (100.0)	-	-	*p* = 0.385	
Transport time, min (*n* = 340)	*p*-value for trend		340 (100.0)	-	-	*p* = 0.753	
Temperature of transportation (*n* = 347)	37 ℃		100 (28.8)	63 (63.0)	37 (37.0)	*p* = 0.133	
	4 ℃		247 (71.2)	176 (71.3)	71 (28.7)		

*Abs* absolute, *BMI* body mass index, *MC* menstrual cycle, *AMH* anti-Mullerian hormone, *IVM* in vitro maturation, *CAPA* capacitation, *BOT* borderline ovarian tumor, *CC* cervical cancer, *EC* e ndometrial cancer, *BC* breast cancer, *OC* ovarian cancer, *HL* Hodgkin lymphoma, *ES* Ewing sarcoma, *PCTs* palliative chemotherapy before the surgery, *AF* antral follicle

The results of the predictors’ identification of the specific types of dysmorphisms are presented in detail in Supplementary Tables 1 and 2. We would like to highlight the most clinically significant results.

Our analyses revealed that the odds of developing cytoplasmic granularity were increased 2.569-fold (95% CI, 1.301–5.179) if the transportation was performed on ice at 4 ℃. The association was also statistically significant in the age-adjustment model (AOR 2.688; 95% CI, 1.314–5.496). At the same time, evaluation of the compared parameters using Cramer’s V showed that this association was weak (*V* = 0.149).

According to the obtained results, the presence of a large polar body was significantly associated with the type of IVM media (*p* = 0.034): the use of CAPA IVM compared to the monophasic standard media reduced the probability of this dysmorphism (OR 0.962; 95% CI, 0.937–0.986; Cramer’s *V* 0.113—weak association).

Statistically significant differences were also found in the probability of small polar body development depending on the age group (*p* = 0.019). Thus, 2.717 times more often (95% CI, 1.195–6.18), this type of dysmorphism developed in patients aged 35 years and older compared to patients in the age range of 18–35 years. The strength of the association between age and the presence of small polar body formation was also weak (*V* = 0.151).

We also found that the development of the dark-color cytoplasm was associated with a history of gynecologic surgery (*p* = 0.002; OR 16.652; 95% CI, 1.977–140.237; Cramer’s *V* 0.187), and these patterns persisted when adjusted for age.

## Discussion

In this study, we explored the frequency of dysmorphism formation in in vitro matured MII oocytes obtained from ovarian tissue. We discovered that at least one type of dysmorphism was present in 69.0% of oocytes. This finding corresponds to the data available on the frequency of oocyte abnormalities in oocytes in IVF cycles [12, 14, 33, 34] but is much lower than previously reported for OTO IVM oocytes in [53]. Thus, our study does not support the hypothesis that in vitro matured ovarian tissue oocytes have better morphology than oocytes after controlled ovarian stimulation in IVF cycles. Nikiforov et al. reported a lower incidence of dysmorphisms of 47% in OTOs, which might be attributed to a different patient population compared to the present research. The patient group was younger with an average age of 29 years (range 17–38 years) compared to the mean age of 31 years (range 14–43) in our study. Additionally, in our study, the majority of patients had gynecological cancers, while in the Nikiforov et al. study, patients had other types of malignancies.

In this work, we demonstrated that ovarian cancer but not the stage of the disease increases the chance of dysmorphism formation by 2.211 times. We can suggest that the factors secreted by a tumor can negatively affect oogenesis. It has been proposed that interleukins and tumor necrosis factors secreted by tumor tissue might contribute to the decline in fertility of cancer patients [54]. In the case of ovarian cancer, the transforming growth factor beta (TGFB) superfamily signaling pathway might play a role, as it promotes ovarian tumorigenesis [55], and at the same time has an impact on granulosa and cumulus cell proliferation and function. Bone morphogenetic proteins (BMPs) and growth differentiation factors (GDFs) as part of the TGF-β superfamily play a vital role in oocyte development and competence acquisition [56].

In our study, we also found that laparoscopic gynecological surgery in the anamnesis was associated with an increase in the frequency of dark-color cytoplasm formation. Laparoscopic procedures involve electrosurgery for tissue dissection and bleeding control, which can have a negative impact on the oogenesis. Decreased follicle number, poorer ovarian response to controlled ovarian hyperstimulation, and decreased live birth rates were previously reported for patients with laparoscopic ovarian surgery due to endometriosis [57–59].

Interestingly, we found that the patient’s age did not influence the majority of oocytes’ morphological characteristics except for the small body formation. This type of dysmorphism was present 2.717 times more often in patients older than 35 years. In the Omidi et al. study on rescue IVM oocytes, the age over 30 years contributed to a more frequent incidence of polar body fragmentation, while there was no statistical difference for any other morphological abnormalities [60]. Fragmentation was the only polar body characteristic that was assessed, and no data on the polar body size was provided in that study.

Notably, in our study, 8 (3.3%) mature oocytes had a smooth endoplasmic reticulum cluster. It corresponds to the data obtained from a large study which reports the 2.8% incidence of SER in MII oocytes after controlled ovarian stimulation [25], but our data contradicts the Nikiforov et al. statement, that SER clusters are absent in oocytes from unstimulated patients undergoing OTO IVM treatment. SER or any other type of dysmorphism can be identified in vitro matured ovarian tissue oocytes, and its formation is most likely not related to controlled ovarian stimulation.

In this study, we demonstrated for the first time that ovarian transportation temperature, but not duration, can influence oocyte quality as the chances of cytoplasmic granularity development increased 2.569 times when transportation occurred on ice. This assumption of the detrimental effect of low temperature during ovarian transportation on the developmental competence of subsequently extracted oocytes was proposed in the ESHRE guideline for female fertility preservation [61]. Transportation of ovarian tissue at low temperatures is beneficial when ovarian tissue cryopreservation (OTC) is planned alongside the OTO IVM as it would slow down the metabolic changes and reduce tissue autolysis in the absence of vascular perfusion [62]. However, at the same time, cumulus-oocyte complexes from antral follicles will be exposed to hypothermia which will lead to cellular and metabolic changes in oocytes. It was demonstrated that storage of different organs and tissues at hypothermic temperatures leads to mitochondrial dysfunction, membrane damage, specifically loss of membrane phospholipids, and intracellular ionic pump activity inhibition [63]. In this study, transportation occurred within one city, ranging from 6 to 75 min. For this short-term transportation, no association was found between the transportation duration and dysmorphism formation. However, it is still unclear how long-term transportation might influence oocyte quality. To sum it up, for OTO IVM cases, we would highly recommend ovarian transportation at 37 ℃, especially for patients with ovarian malignancies. For long-distance transportation and for cases when OTO IVM is combined with OTC, transportation on ice can be considered.

Furthermore, our study indicates that the IVM media might have an impact on oocyte quality as the incidence of large polar body formation was decreased when the biphasic CAPA-IVM system was used. We have previously demonstrated that the use of the CAPA-IVM system leads to a higher maturation rate in ovarian tissue oocytes compared to standard IVM [52] which might be attributed to higher nuclear and cytoplasmic maturation synchrony. Most likely, large polar bodies result from impaired meiosis and premature nuclear maturation. It was demonstrated on a murine model that abnormal meiosis spindle position led to large polar body extrusion [64]. As oocytes with large polar bodies have a decreased potential [65], the selection of IVM media can influence not only the quantity of in vitro mature oocytes but their quality as well. Thus, the application of the biphasic media can be beneficial in OTO IVM programs.

The main limitation of this study is its retrospective and the small number of enrolled patients. Also, the majority of oocytes have not been used for fertilization, and the long-term outcomes of IVM oocytes with different morphological characteristics are not known. The strength of the study is the use of the rather unique data set of in vitro matured ovarian tissue oocytes and the detailed record of the medical histories.

Future studies might implement artificial intelligence technologies for the quality assessment of in vitro matured ovarian tissue oocytes. Several predictive models have recently been developed using AI tools for oocyte quality evaluation. For instance, it has been demonstrated that AI models can identify risks of aneuploidy and poor embryo quality and provide insights into the quality of mature oocytes [66–75]. Predictive models employing AI for oocyte quality evaluation have shown promising results in improving reproductive outcomes [76]. For example, prognostic models such as MAGENTA AI, neural networks, and VIOLET, based on image analysis of mature denuded oocytes, have been demonstrated in several studies to provide significant correlation with subsequent blastocyst development and quality [68, 71–73, 75]. A recent article by Fjeldstad et al. [77] described a deep learning (DL) model for image analysis to assess oocyte quality using static oocyte images and demonstrated that the DL model excels at distinguishing MII oocytes which are likely to lead to a blastocyst from those that would not. Further research on the validation of such AI models on in vitro mature oocytes and especially on ovarian tissue oocytes is needed to utilize machine learning predictions for counseling fertility preservation patients.

## Conclusions

For patients undergoing radical ovariectomies, in vitro matured ovarian tissue oocytes might be the only source of genetic material preservation. Identification of oocyte dysmorphisms and knowing the impact of each dysmorphism on oocyte quality may provide a prognostic tool for evaluating the chances of live birth. In our study, we demonstrated that the frequency of dysmorphisms in in vitro matured oocytes is similar to the oocytes obtained in IVF programs. Both patient and laboratory characteristics might influence the quality of oocytes. Ovarian cancer as a main diagnosis, history of gynecological surgery, and advanced maternal age have a negative impact on the probability of oocyte dysmorphism formation. Transportation of ovaries at 37 ℃ and utilization of biphasic CAPA IVM media might reduce the chance of oocyte abnormalities formation.

## Supplementary Information

Below is the link to the electronic supplementary material.

Supplementary file1 (XLSX 238 KB)

Supplementary file2 (XLSX 17 KB)

## Data Availability

Anonymized raw data will be provided upon request.
